# Supplementary figures and images for: Signal peptidase complex mediates rotavirus VP7 processing and virion assembly
Source: PLoS Pathog. 2026 Mar 20;22(3):e1013688. doi: 10.1371/journal.ppat.1013688 (PMC13043047; doi:10.1371/journal.ppat.1013688)

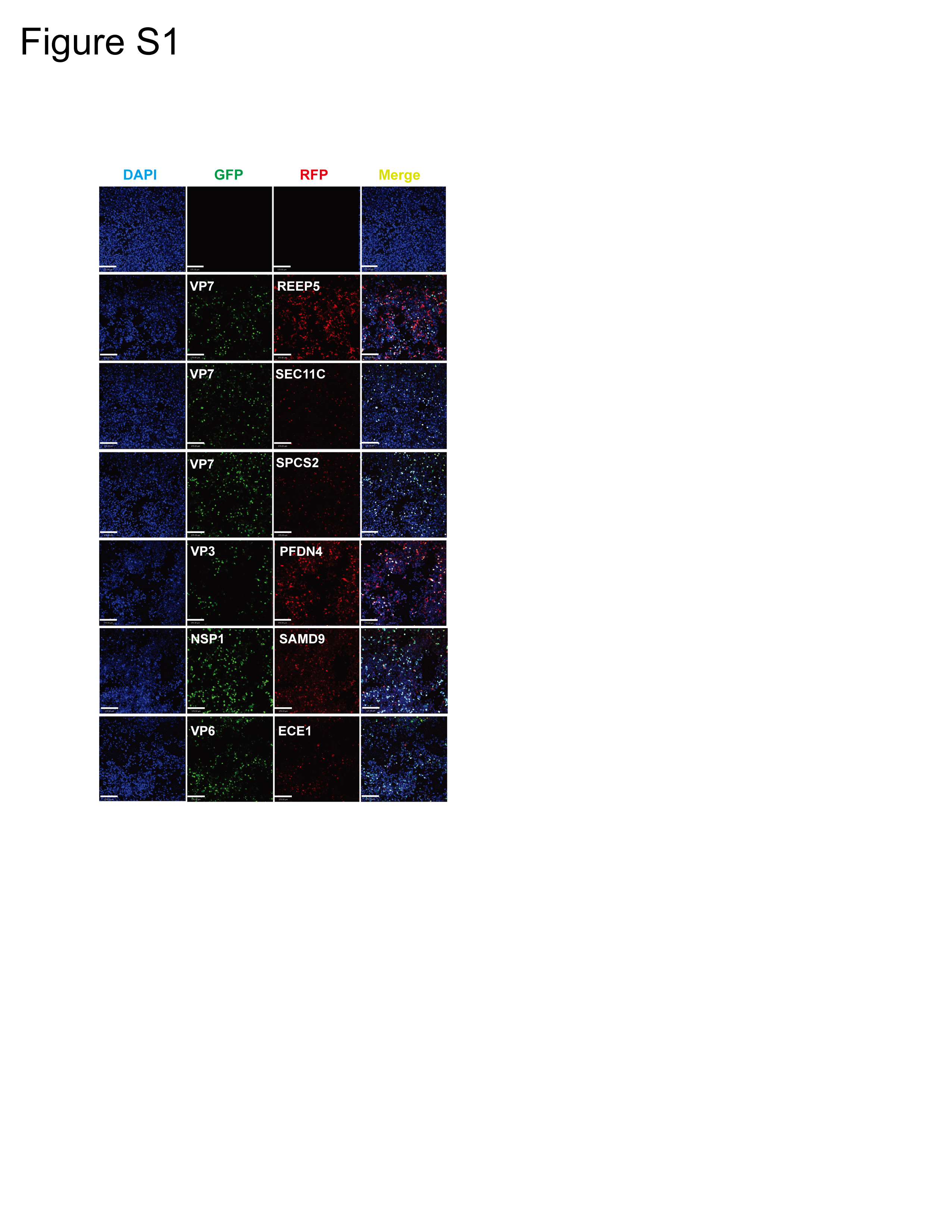

Supplement: S1 Fig — Plasmids expressing -VP7 and REEP5, SEC11C, SPCS2, -VP3 and PFDN4, -NSP1 and SAMD9, -VP6 and ECE1 were co-transfected into HEK293 cells, respectively, and subjected to IFA detection. Viral proteins were tagged with GFP, shown as green fluorescence, while host proteins were tagged with RFP, shown as red fluorescence. Nuclei were counterstained with DAPI. Scale bar, 170 μm. (TIF) [file ppat.1013688.s003.tif]

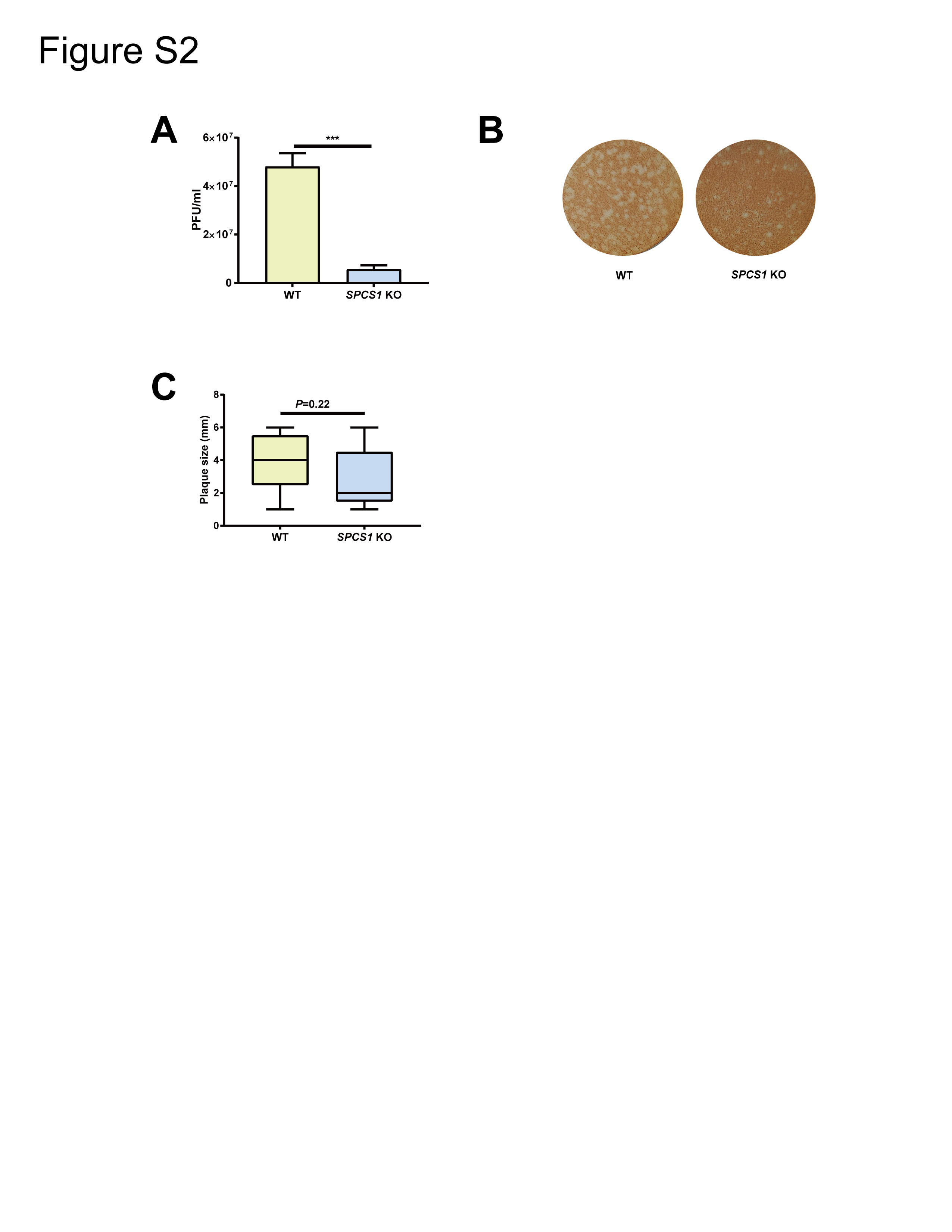

Supplement: S2 Fig — (A)WT and SPCS1 KO HEK293T cells were infected with RRV at an MOI of 3. At 12 hpi, all the cell lysates and supernatants were collected for PFU assays in MA104 to detect the titers. (B) The images of representative plaques from the WT and SPCS1 KO HEK293T cells. Statistical significance was determined by student’ s t comparisons test (***, P < 0.001). (C) The sizes of plaque were measure by Image J from three biological replicates. (TIF) [file ppat.1013688.s004.tif]

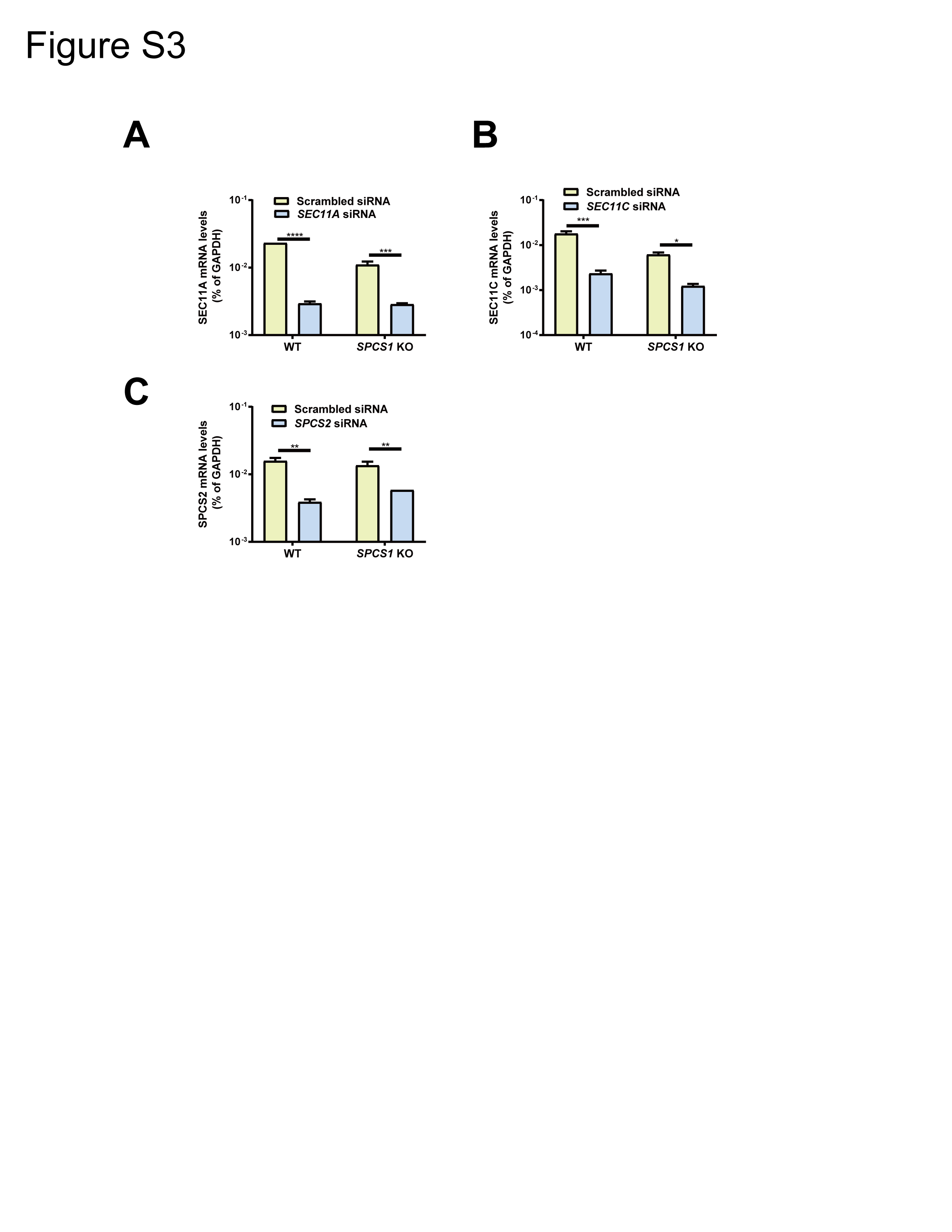

Supplement: S3 Fig — WT and SPCS1 KO HEK293T cells were transfected with siRNAs targeting against SPCS2, SEC11A, SEC11C and a scrambled siRNA at the concentration of 20 nM. At 72 hours post-transfection, cells were collected for RNA extraction, and relative mRNA expression levels were measured by qPCR. The relative expression was normalized to GAPDH. Results are the average of data from two independent experiments and plotted as mean ± SD. Statistical significance was determined by two-way ANOVA with Sidak’s multiple comparisons test (*, P < 0.05, **, P < 0.01; ***, P < 0.001, ****, P < 0.0001). (TIF) [file ppat.1013688.s005.tif]

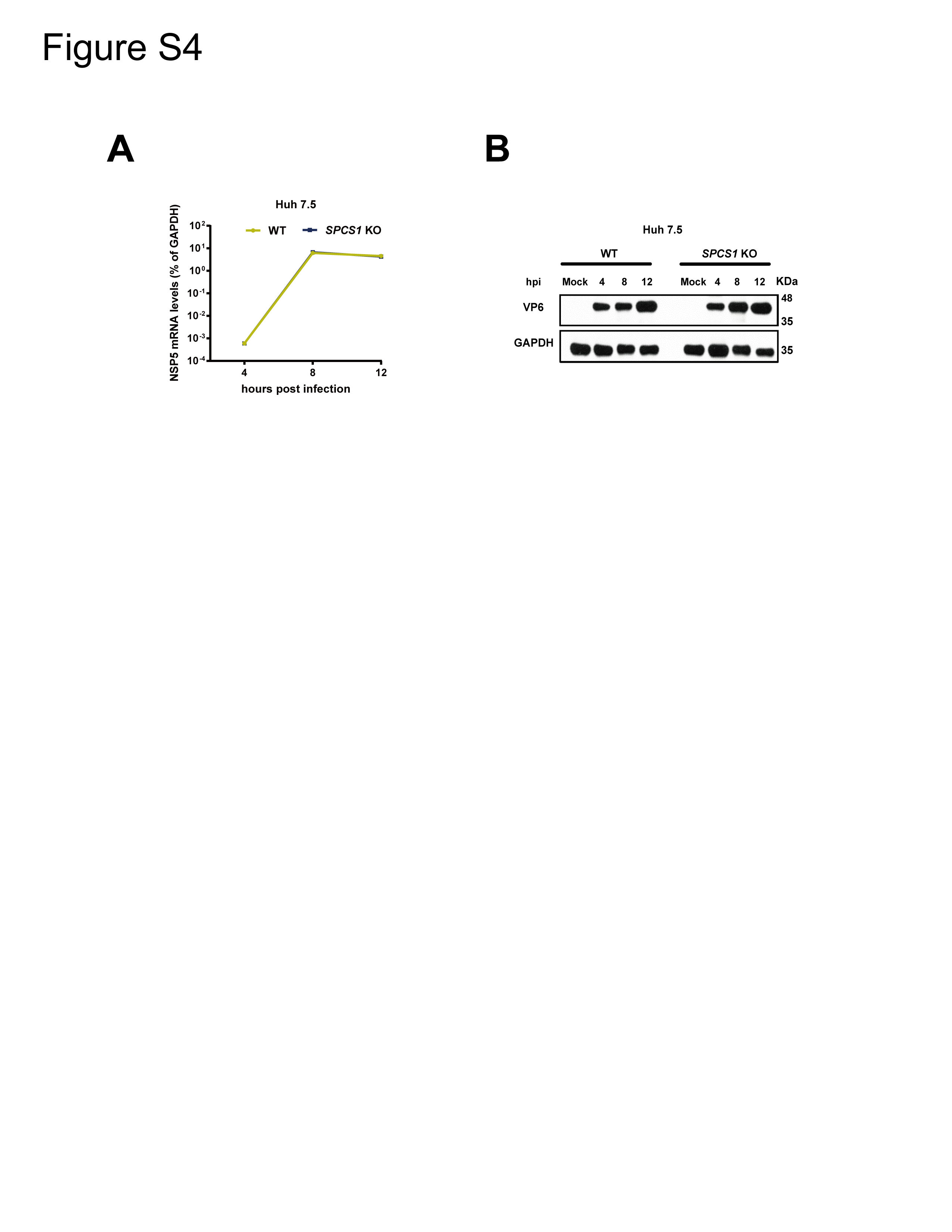

Supplement: S4 Fig — (A) WT and SPCS1 KO Huh7.5 cells were infected with RRV at an MOI of 3. At 4, 8, 12 hpi, the cells were collected for qRT-PCR analysis of viral mRNA level by detecting NSP5. NSP5 level was normalized to GAPDH. The result was representative of one independent experiment. (B) WT and SPCS1 KO Huh7.5 cells were infected with RRV at an MOI of 3. At 4, 8, 12 hpi, the infected and mock cells were harvested for western blot analysis of viral protein levels by detecting VP6. (TIF) [file ppat.1013688.s006.tif]

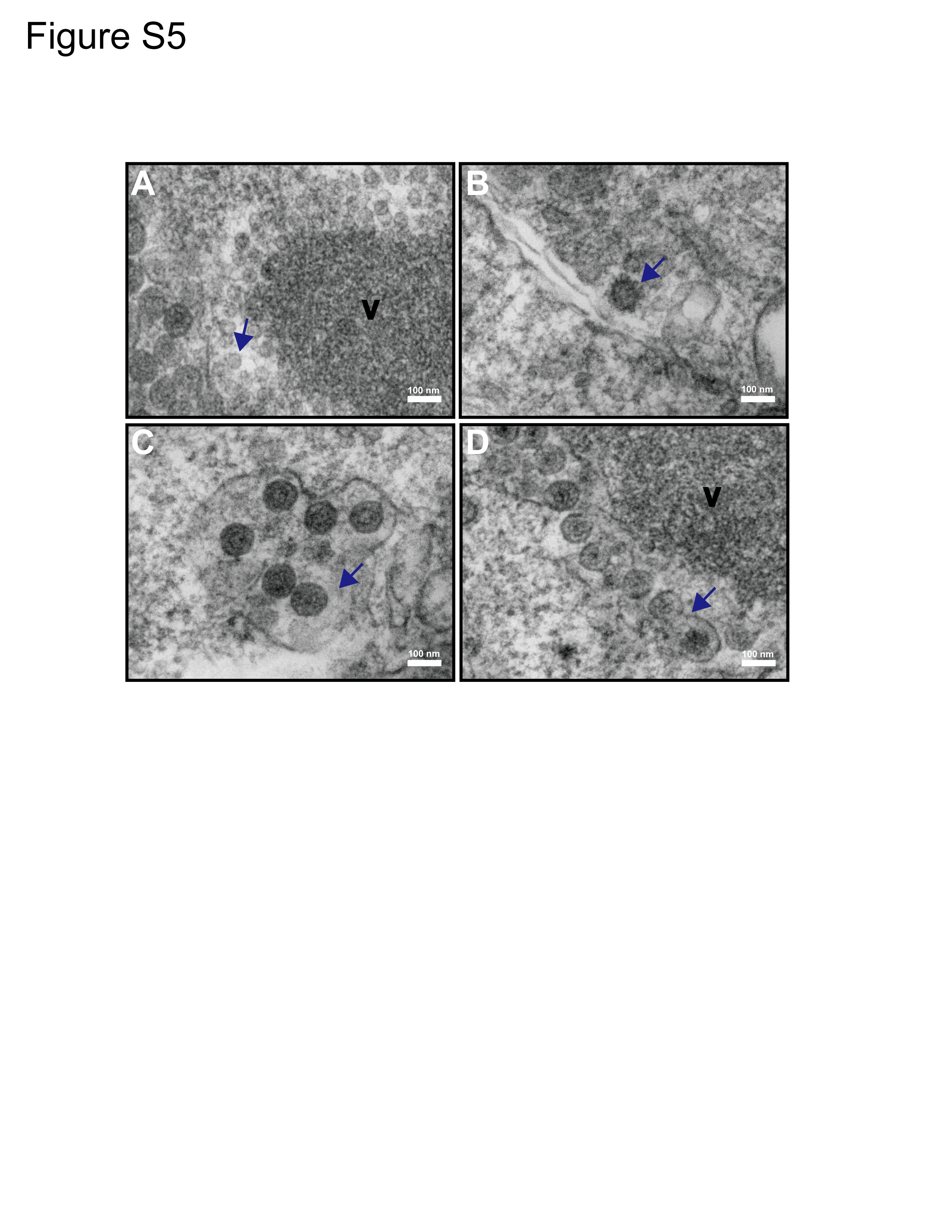

Supplement: S5 Fig — Transmission electron micrographs of RRV particles in HEK293T cells. (A) Single-layered particles indicated by blue arrows surrounding the viroplasm. ‘V’ indicates the viroplasm. (B) DLP, indicated by blue arrow. (C) TLPs, indicated by blue arrow. (D) The budding process of DLP morphing into TL P, indicated by blue arrow, ‘V’ indicates the viroplasm. Scale bar, 100 nm. (TIF) [file ppat.1013688.s001.tif]

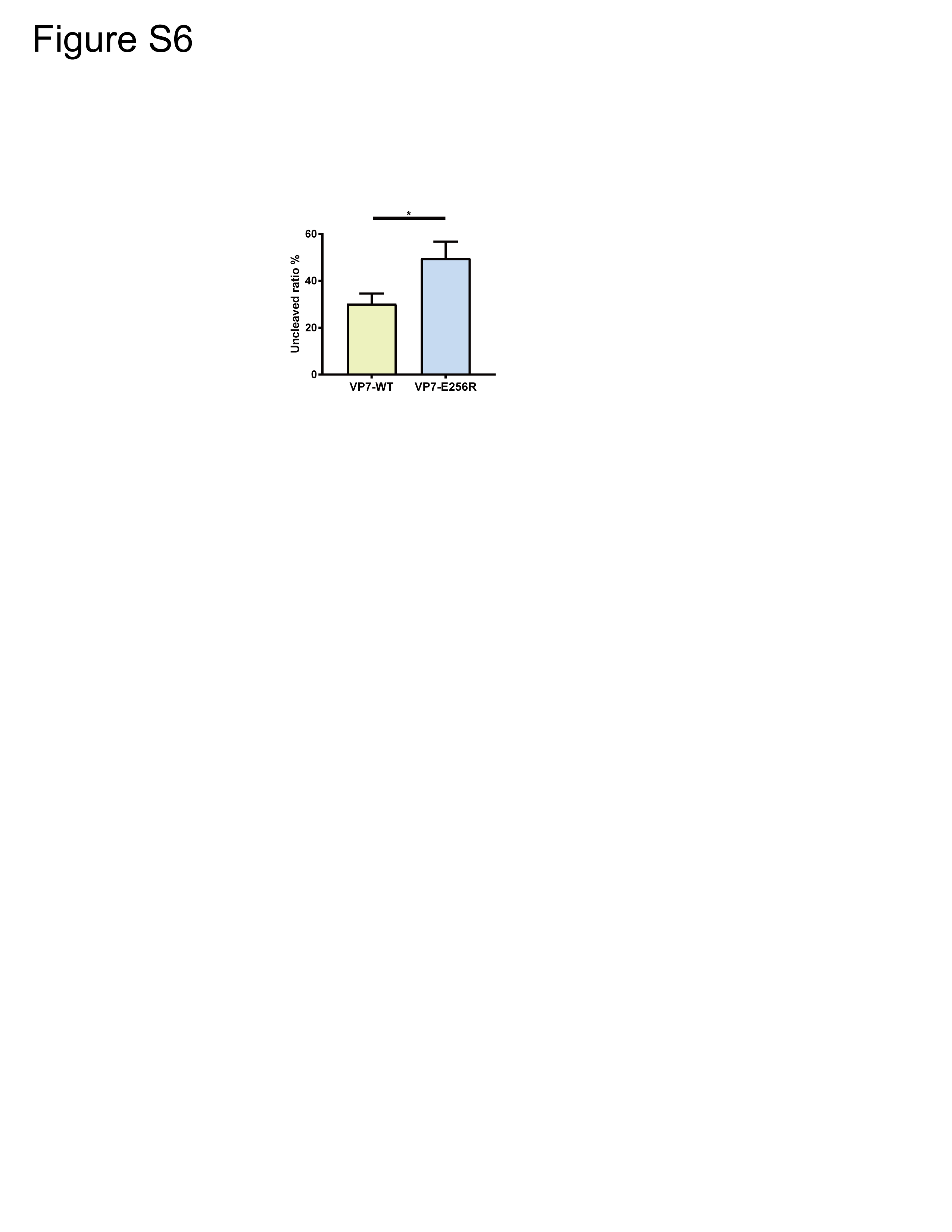

Supplement: S6 Fig — Quantification of the uncleaved ratio of WT and E256R mutant VP7 proteins in HEK293T cells from five independent experiments. The results are the averages of data in five independent experiments and plotted as mean ± SD. Statistical significance was determined by student’s t-test (*, P < 0.05). (TIF) [file ppat.1013688.s007.tif]
